# Complement Receptor 1 Variants Confer Protection from Severe Malaria in Odisha, India

**DOI:** 10.1371/journal.pone.0049420

**Published:** 2012-11-13

**Authors:** Aditya K. Panda, Madhumita Panda, Rina Tripathy, Sarit S. Pattanaik, Balachandran Ravindran, Bidyut K. Das

**Affiliations:** 1 Infectious Disease Biology Group, Institute of Life Sciences, Bhubaneswar, Odisha, India; 2 Department of Biochemistry, S.C.B. Medical College, Cuttack, Odisha, India; 3 Department of Medicine, S.C.B. Medical College, Cuttack, Odisha, India; Lile 2 University, France

## Abstract

**Background:**

In *Plasmodium falciparum* infection, complement receptor-1 (CR1) on erythrocyte’s surface and ABO blood group play important roles in formation of rosettes which are presumed to be contributory in the pathogenesis of severe malaria. Although several studies have attempted to determine the association of CR1 polymorphisms with severe malaria, observations remain inconsistent. Therefore, a case control study and meta-analysis was performed to address this issue.

**Methods:**

Common CR1 polymorphisms (intron 27 and exon 22) and blood group were typed in 353 cases of severe malaria (SM) [97 cerebral malaria (CM), 129 multi-organ dysfunction (MOD), 127 non-cerebral severe malaria (NCSM)], 141 un-complicated malaria and 100 healthy controls from an endemic region of Odisha, India. Relevant publications for meta-analysis were searched from the database.

**Results:**

The homozygous polymorphisms of CR1 intron 27 and exon 22 (TT and GG) and alleles (T and G) that are associated with low expression of CR1 on red blood cells, conferred significant protection against CM, MOD and malaria deaths. Combined analysis showed significant association of blood group B/intron 27-AA/exon 22-AA with susceptibility to SM (CM and MOD). Meta-analysis revealed that the CR1 exon 22 low expression polymorphism is significantly associated with protection against severe malaria.

**Conclusions:**

The results of the present study demonstrate that common CR1 variants significantly protect against severe malaria in an endemic area.

## Background

Malaria, a mosquito borne parasite infection, is caused by various species of genus *Plasmodium: Plasmodium falciparum*, *P. vivax*, *P. ovale*, *P. malariae* and *P. knowlesi*. A recent report of World Health Organization (WHO) estimates about 3.3 billion individuals to be at risk worldwide and reports 216 million cases in 2010 [1]. Infection by *P. falciparum* parasite causes severe disease characterized by cerebral malaria (CM), multi-organ dysfunction (MOD), and non cerebral severe malaria (NCSM), which contributes to high mortality [2], [3]. The pathogenesis of severe malaria remains poorly understood. However, several observations point to multi-factorial mechanisms which includes obstruction of blood vessels by infected RBCs [4] and liberation of inflammatory cytokines [5], [6]. An important phenomenon, rosetting, characterized by adherence of *P. falciparum* infected red blood cells (RBCs) with un-infected RBCs has been presumed to be one of the factors contributing to the development of severe disease [7], [8]. Rosetting is mediated by the parasite ligand, *P. falciparum* erythrocyte membrane protein 1 (PfEMP1) on the surface of infected RBCs, binding to various molecules like complement receptor 1 (CR1/CD35) [9] and blood groups antigens [2]. CR1 seems to play an important role in the pathogenesis of severe malaria by virtue of its capacity to rosette, form immune complexes (ICs) and facilitate entry of parasite into the RBCs [10]. Beside rosetting, CR1 plays an important role in regulation of complement activation. It acts as a co-factor by binding to complement cleavage products C3b and C4b and produce the inactive form of complements iC3b and iC4b, respectively [11]. Individuals with higher CR1 expression are believed to develop severe pathology like CM as a result of enhanced rosette formation leading to blockage of blood flow in brain capillaries [9]. On the other hand, lower levels of CR1 on erythrocyte surface are associated with severe anemia [12]. However, literature indicates that endemicity plays a role in CR1 functionality [13], [14]. In high endemic areas CR1 expression remains low compared to non-endemic areas [13], [14].

CR1 is a membrane glycoprotein expressed on wide range of cells viz. red blood cells (RBCs), monocytes, granulocytes, glomerular podocytes, follicular dendritic cells, all B cells and some T cells [15], [16]. CR1 expression on RBCs surface varies between individuals in the range of 50–1,200 molecules per cell [17]. The differential surface expression has been attributed to various single nucleotide polymorphisms (SNPs) in CR1 genes. A transversion mutation (A>T) in intron27 (rs11118133) has been correlated with reduced CR1 levels on RBCs in Thai [18], Caucasians [17] and Indian population [13], [19] but not in Africans [20], [21]. Another well investigated SNP, transition of A to G in exon 22 (rs2274567), has also been associated with lower CR1 expression in Caucasian [14], [22], residents of Papua New Guinea [14], and Indians [13], [19]. However, levels of CR1 expression and its functionality related to disease severity has not been unequivocally concordant [10]. Intron 27 variant is associated with susceptibility to coronary artery disease, dyslipidemia [23], sarcoidosis [24] and idiopathic pulmonary fibrosis [25]. Although, exon 22 polymorphism correlates with CR1 exprassion [13], [19], it fails to any show association with the above disorders [25], [26], and gallbladder cancer [27].

**Table 1:** Details of study participants.

Subjects	Severe *P. falciparum* malaria clinical categories (SM)					P value
	UM	CM	MOD	NCSM	HC	
**Total number (n)**	141	97	129	127	100	NA
**Sex (M/F)**	106/35	71/26	103/26	104/23	71/29	NA
**Mean age in years (range)**	34.48 (15–80)	33.78 (15–86)	34.59 (15–83)	34.79 (15–70)	31.18 (15–72)	NS
**Mean Hb (g/dl)±SD**	10.70±1.94	9.87±2.02	9.84±2.17	9.70±2.46	12.09±1.91	**<0.0001**

Note: Data are no. (%) of participants unless otherwise specified. UM; uncomplicated malaria, CM; cerebral malaria, MOD; multiorgan dysfunction, NCSM; non cerebral severe malaria, HC; healthy controls, NA; not applicable, NS; not significant.

The role of several common single nucleotide polymorphisms (SNPs) in severe *P. falciparum* malaria has been investigated earlier [10]. Most of the genetic association studies were focused on intron 27 and exon 22 polymorphisms. Thus in the current study we performed a case control study and meta-analysis to understand the role of intron 27 and exon 22 in *P. falciparum* malaria. The association of intron 27 polymorphism with severe malaria has been contradictory. The CR1 low expression genotype (TT) has been shown to be associated with protection against cerebral malaria in an eastern Indian cohort [19]. While in a study on a Thai population, found that the low CR1 TT genotype was associated with increased risk of severe disease [18]. The role of exon 22 polymorphism has also remained controversial. A study from eastern India showed protection of homozygous mutant (GG) from cerebral malaria [19], on the contrary, in a non-endemic population GG individuals were susceptible to severe disease [13]. The heterozygous (AG) of exon 22 polymorphism provided protection in Papua New Guinea [14]. However, an independent study in Thailand and Myanmar border failed to show any association of both intron 27 and exon 22 polymorphisms with *P. falciparum* infection [28].

Blood group is another important factor determining rosetting capacity of infected RBCs and has been associated with pathophysiology of severe *P. falciparum* malaria [29]. Blood group ‘O’ is correlated with lower rosetting capacity [29] and protection [30]. Association of other blood groups with rosetting and their role in disease susceptibility has been population specific [31]. Recently we demonstrated that blood group ‘B’ is associated with susceptibility and ‘O’ with protection against severe malaria in Odisha, India [2].

The observed relationship between CR1 polymorphisms and severe malaria remains inconsistent. Since previous studies were under powered, we conducted a tertiary care hospital based case-control study comprising of large number of patients to probe this relationship. A meta-analysis was further performed to summarize the earlier observed data (Supplement S1). Since CR1 and ABO blood group system are both responsible for rosetting, we hypothesized that combined effect may be more significant.

**Table 2:** Genotype and allele of CR1 (exon 22 and intron 27) polymorphisms and blood groups distribution in *P. falciparum* malaria.

SNPs		Clinical categories, % of subject					CM Vs UM		MOD Vs UM		NCSM Vs UM		SM Vs UM	
CR1 exon 22	CM(n = 97)	MOD(n = 129)	NCSM(n = 127)	SM(n = 353)	UM(n = 141)	HC(n = 100)	OR (95% CI)	P Value	OR (95% CI)	P Value	OR (95% CI)	P value	OR (95% CI)	P value
**AA**	31 (32)	40 (31)	23 (18)	94(27)	17 (12)	15 (15)	1	Ref	1	Ref	1	Ref	1	Ref
**AG**	39 (40)	59 (46)	54 (42)	152(43)	56 (40)	41 (41)	0.38 (0.18 to 0.78)	0.01	0.37 (0.18 to 0.73)	0.004	0.71 (0.34 to 1.47)	0.46	0.49 (0.26 to 0.89)	0.02
**GG**	27 (28)	30 (23)	50 (40)	107(30)	68 (48)	44 (44)	**0.21 (0.10 to 0.45)**	**<0.0001**	**0.18 (0.09 to 0.38)**	**<0.0001**	0.54 (0.26 to 1.12)	0.10	**0.28 (0.15 to 0.51)**	**<0.0001**
**A**	101 (52)	139(54)	100 (39)	340(48)	90 (32)	71 (35.5)	1	Ref	1	Ref	1	Ref	1	Ref
**G**	93 (48)	119 (46)	154 (61)	366(52)	192 (68)	129 (64.5)	0.43 (0.29 to 0.62)	**<0.0001**	0.40 (0.28 to 0.56)	**<0.0001**	0.72 (0.50 to 1.03)	0.08	**0.50 (0.37 to 0.67)**	**<0.0001**
**CR1 intron 27**	CM(n = 97)	MOD(n = 129)	NCSM(n = 127)	SM(n = 353)	UM(n = 141)	HC(n = 100)								
**AA**	49 (51)	59 (46)	31 (25)	139(39)	27 (20)	22 (22)	1	Ref	1	ref	1	Ref	1	Ref
**AT**	33 (34)	49 (38)	59 (46)	141(40)	70 (50)	51 (51)	**0.25 (0.13 to 0.48)**	**<0.0001**	0.32 (0.17 to 0.57)	**0.0001**	0.71 (0.38 to 1.32)	0.34	**0.39 (0.23 to 0.64)**	**0.0002**
**TT**	15 (15)	21 (16)	37 (29)	73(21)	44 (30)	27 (27)	**0.18 (0.08 to 0.39)**	**<0.0001**	0.21 (0.10 to 0.43)	**<0.0001**	0.70 (0.36 to 1.39)	0.39	**0.32 (0.18 to 0.56)**	**<0.0001**
**A**	131 (67)	167 (65)	121 (48)	419(59)	124 (44)	95 (47.5)	1	Ref	1	ref	1	Ref		
**T**	63 (33)	91 (35)	133 (52)	287(41)	158 (56)	105 (52.5)	**0.37 (0.25 to 0.55)**	**<0.0001**	**0.42 (0.30 to 0.60)**	**<0.0001**	0.86 (0.61 to 1.21)	0.43	**0.53 (0.40 to 0.71)**	**<0.0001**
**Blood group**	CM(n = 75)	MOD(n = 93)	NCSM(n = 101)	SM(n = 269)	UM(n = 119)	HC(n = 100)								
**O^+^**	16 (21)	24 (26)	32 (32)	72(27)	53 (45)	46 (46)	1	Ref	1	ref	1	Ref	1	
**A^+^**	9 (12)	19 (20)	18 (18)	46(17)	26 (22)	21 (21)	1.14 (0.44 to 2.94)	0.81	1.61 (0.75 to 3.46)	0.24	1.14 (0.54 to 2.41)	0.84	0.76(0.42 to 1.39)	0.45
**B^+^**	42 (56)	45 (49)	44 (43)	131(49)	22 (18)	22 (22)	**6.32 (2.95 to 13.53)**	**<0.0001**	**4.51 (2.23 to 9.11)**	**<0.0001**	**3.31 (1.68 to 6.50)**	**0.0005**	**4.38 (2.46 to 7.78)**	**<0.0001**
**AB^+^**	8 (11)	5 (5)	7 (7)	20(7)	18 (15)	11 (11)	1.47 (0.53 to 4.01)	0.44	0.61 (0.20 to 1.84)	0.44	0.64 (0.24 to 1.71)	0.47	1.22 (0.58 to 2.53)	0.70
**Non O (A^+^+ B^+^ + AB^+^)**	**59 (79)**	69 (74)	69 (68)	197(73)	66 (55)	54 (54)	**2.96 (1.53 to 5.73)**	**0.001**	**2.30 (1.28 to 4.16)**	**0.006**	1.73 (0.99 to 3.01)	0.05	**2.19(1.39 to 3.45)**	**0.0009**

NOTE: Data are no. (%) of participants unless otherwise specified. UM: uncomplicated malaria; CM: cerebral malaria; MOD: multi-organ dysfunction; NCSM: non-cerebral severe malaria; OR: odds ratio; CI: confidence interval.

## Materials and Methods

### Ethics Statements

The study and its protocols was approved by the Institutional Human Ethics Committee of S.C.B. Medical College Cuttack. Blood samples were collected after written consent of the healthy controls and patients or accompanying person (in case of comatose patients).

### Study Site and Sample Collection

The study was conducted at SCB Medical College and Hospital, Cuttack, Odisha. The state is considered endemic for malaria with more than 85% cases attributed to *P. falciparum* [32]. Patients enrolled in the current study belonged to the coastal districts having an average annual parasite index (API) of 6.67 [33]. Clinically suspected malaria patients were subjected to immune chromatography test (SD Bio Standard Diagnostics India) and Giemsa-stained thick and thin blood smears. Patients who were positive for ICT but negative by blood smears were subjected to nested polymerase chain reaction (PCR) [2] for confirmation of *P. falciparum* infection. *P. falciparum* infected individuals were categorized based on WHO guidelines. Uncomplicated malaria (UM) was defined as patients with fever and positive for *P. falciparum* infection. Severe malaria (SM) was categorized into three groups based on patients clinical features: 1) Cerebral malaria (CM), 2) Non cerebral severe malaria (NCSM) and 3) Multi-organ-dysfuction (MOD). CM was further defined as patients with altered sensorium, GCS (Glasgow Coma Scale) of ≤10. NCSM patients had one of the several manifestations of severe malaria without cerebral involvement, namely severe anaemia (haemoglobin <5 g/dl), acute renal failure (serum creatinine >3 mg/dl), jaundice (serum bilirubin >3 mg/dl), acute respiratory distress syndrome (PaO2/FIO2<200), haemoglobinuria (dark red or black colored urine positive for haemoglobin) and shock (systolic BP of <80 mm Hg). MOD was diagnosed based on presence of two or more organ involvement like CNS (GCS≤10), respiratory (PaO2/FIO2<200), renal failure (serum creatinine >3 mg/dl) and hepatic dysfunction (ALT/AST >3 times of normal, prolonged prothrombin time and albuminaemia) [2], [3]. Patients with following criteria were excluded from current investigations: i) Co-infection with other *Plasmodium* species, ii) chronic disease like tuberculosis, chronic renal failure, cirrhosis of liver and autoimmune diseases like systemic lupus erythematosus and rheumatoid arthritis. 100 healthy controls (HC) from a similar geographical background were enrolled. None of the healthy reported history of clinical malaria in the last 5 years. They were essentially healthy and negative for *P. falciparum* infection. The risk of exposure to malaria was similar for both HC and patients. *P. falciparum* infected patients and HC were not related to each other. About 5 ml of venous blood was collected in EDTA vials from all enrolled patients.

**Table 3:** Haplotype analysis of CR1polymorphisms in *P. falciparum* malaria patients.

CR1 haplotypes	Clinical categories					CM Vs UM		MOD Vs UM		NCSM Vs UM		SM vs UM	
exon 22/intron 27	UM (n = 141)	CM (n = 97)	MOD (n = 129)	NCSM (n = 127)	SM (n = 353)	OR (95% CI)	P Value	OR (95% CI)	P Value	OR (95% CI)	P value	OR (95% CI)	P value
**G-T**	51.33	30.12	29.75	46.06	35.41	1	Ref	1	Ref	1	Ref	1	Ref
**A-A**	27.22	49.70	48.35	33.46	43.90	**3.03 (1.66 to 5.53)**	**0.0003**	**3.01 (1.71 to 5.28)**	**0.0001**	1.38 (0.79 to 2.40)	0.26	**2.35 (1.48 to 3.72)**	**0.0003**
**G-A**	16.75	17.82	16.38	14.57	15.86	1.70 (0.80 to 3.61)	0.17	1.61 (0.79 to 3.26)	0.20	0.91 (0.45 to 1.84)	0.85	1.41 (0.80 to 2.48)	0.33
**A-T**	4.69	2.36	5.52	5.91	4.81	0.68 (0.13 to 3.49)	1.00	1.84 (0.60 to 5.64)	0.37	1.22 (0.40 to 3.67)	0.78	1.64 (0.62 to 4.36)	0.65

NOTE: Data are shown in percentage. UM: uncomplicated malaria; CM: cerebral malaria; MOD: multi-organ dysfunction; NCSM: non-cerebral severe malaria; OR: odds ratio; CI: confidence interval.

### DNA Isolation and Genotyping of CR1 Variants

DNA was isolated from whole blood by QIAamp DNA Blood Mini Kit (QIAGEN). Samples were genotyped for intron27 HindIIIA>T, exon22 3650A>G, gene by PCR-RFLP as described earlier [22]. For intron27 and exon22 the RFLP products were separated on 3.5% neusive agarose and visualized by ethidium bromide staining under trans UV illumination. For confirmation of genotyping results, about 20% of the randomly selected samples were directly sequenced and the results were observed to be 100% concordant.

### Blood Group Typing

Blood group of *P. falciparum* infected patients (n = 388) and healthy controls (n = 100) were typed by commercial haemagglutination kit (Tulip Diagnostics, Goa, India).

**Table 4:** Association of CR1 polymorphisms, ABO blood group in treatment outcome patients with severe malaria.

Markers	Dead	Survivors	P value, OR (95%CI)
CR1 exon 22	(n = 44)	(n = 450)	
AA	20 (46)	91 (20)	Ref, 1
AG	16 (36)	192 (43)	**0.008**, 2.63 (1.30 to 5.32)
GG	8 (18)	167 (37)	**0.0003**, 4.58 (1.94 to 10.83)
A	56 (64)	374 (42)	Ref, 1
G	32 (36)	526 (58)	**0.0001**, 2.46 (1.56 to 3.87)
CR1 intron 27	(n = 44)	(n = 450)	
AA	27 (61)	140 (31)	Ref, 1
AT	13 (30)	197 (44)	**0.002**, 2.92 (1.45 to 5.86)
TT	4 (9)	113 (25)	**0.0005**, 5.44 (1.85 to 16.03)
A	67 (76)	477 (53)	Ref, 1
T	21 (24)	423 (47)	**<0.0001**, 2.82 (1.70 to 4.70)
Blood groups	(n = 30)	(n = 358)	
O^+^	6 (20)	119 (33)	Ref, 1
A^+^	6 (20)	65 (18)	0.35, 0.54 (0.16 to 1.76)
B^+^	17 (57)	137 (39)	0.07, 0.40 (0.15 to 1.06)
AB^+^	1 (3)	37 (10)	1.00, 1.86 (0.21 to 16.01)
Non O (A^+^+ B^+^ + AB^+^)	24 (80)	239 (67)	0.15, 0.50 (0.19 to 1.26)

NOTE: Data are no. (%) of participants unless otherwise specified. CM: cerebral malaria; MOD: multi-organ dysfunction; NCSM: non-cerebral severe malaria; UM: uncomplicated malaria; OR: odds ratio; CI: confidence interval.

**Table 5:** Haplotype analysis of CR1polymorphisms in treatment outcome of *P. falciparum* malaria patients.

CR1 haplotypes	Survivors (n = 450)	Dead (n = 44)	P value, OR (95%CI)
G-T	42.06	21.26	ref, 1
A-A	36.62	61.03	**0.0007, 0.29 (0.14 to 0.59)**
G-A	16.38	15.11	0.19, 0.52 (0.20 to 1.30)
A-T	4.7	2.61	1.00, 0.91 (0.18 to 4.48)

NOTE: Data are shown in percentage. OR: odds ratio; CI: confidence interval.

### Publication Search and Data Extraction

An extensive search was performed by two authors AKP and MP on Pubmed and Science direct with a combination of following key words: “CR1”, “polymorphism”, “falciparum” and “malaria”. To find out additional relevant reports, references of original studies were screened thoroughly. Eligible studies included in this meta-analysis supported the following criteria: (a) association of intron 27 and/or exon 22 polymorphism and severe malaria (b) use of a case-control protocol (c) genotypes frequencies or Odds ratio (OR) and 95% confidence interval (CI) values and (d) published in English language.

**Table 6:** Association of combined blood groups and CR1 polymorphisms (intron 27 and exon 22) with *P. falciparum* malaria.

Combined		Clinical categories, % of subject				CM vs UM		MOD vs UM		NCSM vs UM		SM vs UM	
Blood group/intron 22/exon 27	CM(n = 75)	MOD(n = 93)	NCSM(n = 101)	SM(n = 269)	UM (n = 119)	OR(95% CI)	P Value	OR (95% CI)	P Value	OR (95% CI)	P value	OR (95% CI)	P value
O^+^/AA/AA	3 (4)	3 (3)	3 (3)	9 (3)	6 (5)	1	Ref	1	ref	1	Ref		ref
O^+^/AA/AT+TT	1 (1)	1 (1)	2 (2)	4 (1.5)	2 (2)	1.00 (0.06 to16.00)	1.00	1.00 (0.06 to16.00)	1.00	2.00 (0.18 to 22.07)	1.00	1.33 (0.18 to 9.73)	1
O^+^/AG+GG/AA	3 (4)	6 (6.5)	2 (2)	11 (4)	3 (3)	2.00 (0.24 to 16.62)	0.62	4.00 (0.56 to 28.41)	0.34	1.33 (0.13 to 12.83)	1.00	2.44 (0.47 to 12.63)	0.42
O^+^/AG+GG/AT+TT	9 (12)	14 (15)	25 (25)	48 (18.5)	42 (34)	0.42 (0.08 to 2.04)	0.36	0.66 (0.14 to 3.02)	0.68	1.19 (0.27 to 5.18)	1.00	0.76 (0.25 to 2.31)	0.78
A^+^/AA/AA	1 (1)	4 (4)	1 (1)	6 (2)	3 (3)	0.66 (0.04 to 9.47)	1.00	2.66 (0.34 to 20.52)	0.61	0.66 (0.04 to 9.47)	1.00	1.33 (0.23 to 7.51)	1.
A^+^/AA/AT+TT	0	2 (2)	0	2 (1)	0	–	–	–	–	–	–	–	–
A^+^/AG+GG/AA	1 (1)	6 (6.5)	2 (2)	9 (3)	4 (3)	0.50 (0.03 to 6.68)	1.00	3.00 (0.45 to 19.60)	0.36	1.00 (0.11 to 8.95)	1.00	1.50 (0.31 to 7.18)	0.70
A^+^/AG+GG/AT+TT	7 (9)	7 (8)	15 (15)	29 (11)	18 (14.5)	0.77 (0.15 to 4.00)	1.00	0.77 (0.15 to 4.00)	1.00	1.66 (0.35 to 7.82)	0.70	1.07 (0.32 to 3.52)	1.00
B^+^/AA/AA	17 (24)	17 (18)	8 (8)	42 (15.5)	2 (2)	**17.00 (2.26 to 127.8)**	**0.004**	**17.00 (2.26 to 127.8)**	**0.004**	8.00 (1.00 to 64.00)	0.06	**14.00 (2.42 to 80.99)**	**0.002**
B^+^/AA/AT+TT	1 (1)	1 (1)	1 (1)	3 (1)	1 (1)	2.00 (0.09 to 44.38)	1.00	2.00 (0.09 to 44.38)	1.00	2.00 (0.09 to 44.38)	1.00	2.00 (0.16 to 24.08)	1.00
B^+^/AG+GG/AA	9 (12)	7 (8)	6 (6)	22 (8)	2 (2)	9.00 (1.14 to 71.07)	0.06	7.00 (0.86 to 56.92)	0.15	6.00 (0.72 to 49.86)	0.15	7.33 (1.23 to 43.43)	0.037
B^+^/AG+GG/AT+TT	15 (20)	20 (22)	29 (28)	64 (24)	18 (14.5)	1.66 (0.35 to 7.82)	0.70	2.22 (0.48 to 10.22)	0.46	3.22 (0.71 to 14.53)	0.15	2.37 (0.74 to 7.54)	0.19
AB^+^/AA/AA	2 (3)	0	1 (1)	3 (1)	0	–	–	–	–	–	–	–	–
AB^+^/AA/AT+TT	0	0	0	0	1 (1)	–	–	–	–	–	–	–	–
AB^+^/AG+GG/AA	3 (4)	0	1 (1)	4 (1.5)	3 (3)	2.00 (0.24 to 16.62)	0.62	–	–	0.66 (0.04 to 9.47)	1.00	0.88 (0.14 to 5.48)	1.00
AB^+^/AG+GG/AT+TT	3 (4)	5 (5)	5 (5)	13 (5)	14 (12)	0.42 (0.06 to 2.76)	0.62	0.71 (0.12 to 3.99)	1.00	0.71 (0.12 to 3.99)	1.00	0.61(0.17 to 2.22)	0.53

NOTE: Data are no. (%) of participants unless otherwise specified. UM: uncomplicated malaria; CM: cerebral malaria; MOD: multi-organ dysfunction; NCSM: non-cerebral severe malaria; OR: odds ratio; CI: confidence interval.

### Statistical Analysis

A power calculation was performed a priori using PS: Power and Sample Size Calculation Software (http://biostat.mc.vanderbilt.edu/twiki/bin/view/Main/PowerSampleSize) [34], which showed that a sample size of at least 200 patients with severe malaria and 200 uncomplicated malaria would provide a power of 80% to detect an increase in the relative risk of developing severity of at least 2.3 with a 2-tailed α = 0.05 (type 1 error probability of 5%). Genotype and allele frequency were calculated by direct counting. SNPalyze software (Dynacom, Japan) was employed to calculate Hardy-Weingberg equilibrium. Fisher’s test was used for comparison of genotype, allele frequencies and to test association of combined genotype distribution among various clinical categories. Odds ratios (ORs), 95% confidence intervals (95% CIs) were calculated by Graphpad prism 5.01. P value less than 0.01 was taken as significant (Bonferroni correction for three markers 0.05/3 = 0.01).

All statistical analysis for meta-analysis was performed by comprehensive meta-analysis (CMA) V2 software. The association of intron 27 and exon 22 with severe malaria was assessed by calculation of OR and 95% CI in various models: (i) the allele contrast (intron 27: T versus A; exon 22: G versus A), (ii) homozygous comparison (intron 27: TT vs AA; exon 22: GG vs AA), (iii) heterozygous comparison (intron 27: AT vs AA; exon 22: GA vs AA), (iv) the dominant model (intron 27: TT+AT versus AA; exon 22: GG+AG versus AA) and (v) recessive model (intron 27: TT versus AA+AT; exon 22: GG versus AA+AG). Heterogeneity between the studies was tested by chi-square based Cochran’s Q statistic. A significant *P* value (<0.10) indicates heterogeneity among studies and combined OR was calculated by random effect model (the Mantel-Haenszel method) [35]. In contrast, fixed effect model (the Der Simonian and Laird method) [36] was used for calculation of combined OR for homogeneity among studies. In addition, I^2^ statistics was used to quantify inter study variability. It ranges between 0% and 100%, where a value of 0% indicates no observed heterogeneity, and larger values indicate an increasing degree of heterogeneity [37]. The funnel plot was employed to examine the publication bias. Egger’s regression analysis was used for re-evaluation of publication bias, and *P* value less than 0.10 was considered to be significant.

**Table 7:** Characteristics of all studies included in meta-analysis.

Authors, Year, Ref	Country	SNPs	Genotyping Method	Numbers (SM/MM)	SM			MM			Association with severe malaria	Functional relevance
					AA/AA	AT/AG	TT/GG	AA/AA	AT/AG	TT/GG		
Nagayasu et al. 2001, [18]	Thailand	rs11118133	PCR-RFLP	55/130	17 (31)	19 (34.5)	19 (34.5)	41 (32)	68 (52)	21 (16)	TT genotype is associated with SM	Low CR1 expression in TT genotype
Cockburn et al. 2004, [14]	PNG	rs2274567	PCR-RFLP	180/178	21 (12)	57 (32)	102 (56)	9 (5)	81 (45)	88 (50)	AG genotype conferred protection from SM	AA genotype is associated with high CR1 expression.
Teeranaipong et al. 2008, [28]	Thailand	rs11118133	PCR-RFLP	272/203	68 (25)	135 (50)	69 (25)	55 (27)	104 (51)	44 (22)	NA	NA
		rs2274567	PCR-RFLP	274/203	70 (26)	136 (50)	68 (24)	44 (22)	105 (52)	54 (26)	NA	ND
Sinha et al. 2009, [13]	India (non-endemic)	rs11118133	PCR-RFLP	41/12	17 (41.5)	7 (17)	17 (41.5)	5 (42)	4 (33)	3 (25)	AA protect from SM	High CR1 expression in AA genotype
		rs2274567	SNaPShot	65/10	16 (25)	23 (35)	26 (40)	2 (20)	7 (70)	1 (10)	Allele G and GG genotype are associated with SM	High CR1 expression in AA genotype
Sinha et al. 2009, [13]	India (endemic)	rs11118133	PCR-RFLP	25/76	1 (4)	11 (44)	13 (52)	25 (33)	28 (37)	23 (30)	NA	High CR1 expression in AA genotype
		rs2274567	SNaPShot	23/76	6 (26)	10 (43)	7 (31)	23 (31)	33 (43)	20 (26)	NA	High CR1 expression in AA genotype
Rout et al. 2011, [19]	India	rs11118133	PCR-RFLP	145/210	38 (26)	64 (44)	43 (30)	42 (20)	107 (51)	61 (29)	AA genotype is associated with CM, TT with SMA	High CR1 expression in AA genotype
		rs2274567	PCR-RFLP	145/210	29 (20)	66 (45.5)	50 (34.5)	23 (11)	85 (40)	102 (49)	AA genotype is associated with CM, GG with SMA	High CR1 expression in AA genotype
Current study	Odisha, India	rs11118133	PCR-RFLP	353/141	94 (27)	152 (43)	107 (30)	17 (12)	56 (40)	68 (48)	AA genotype susceptible to CM & MOD	ND
		rs2274567	PCR-RFLP	353/141	139 (39)	141 (40)	73 (21)	27 (20)	70 (50)	44 (30)	AA susceptible to CM & MOD	ND

NOTE: Data are no. (%) of participants unless otherwise specified. SM; severe malaria, MM; Mild malaria, SMA; severe malaria anemia, PNG; Papua New Guinea, RFLP; restriction fragment length polymorphisms, NA; not associated, ND; not done.

**Figure 1:**
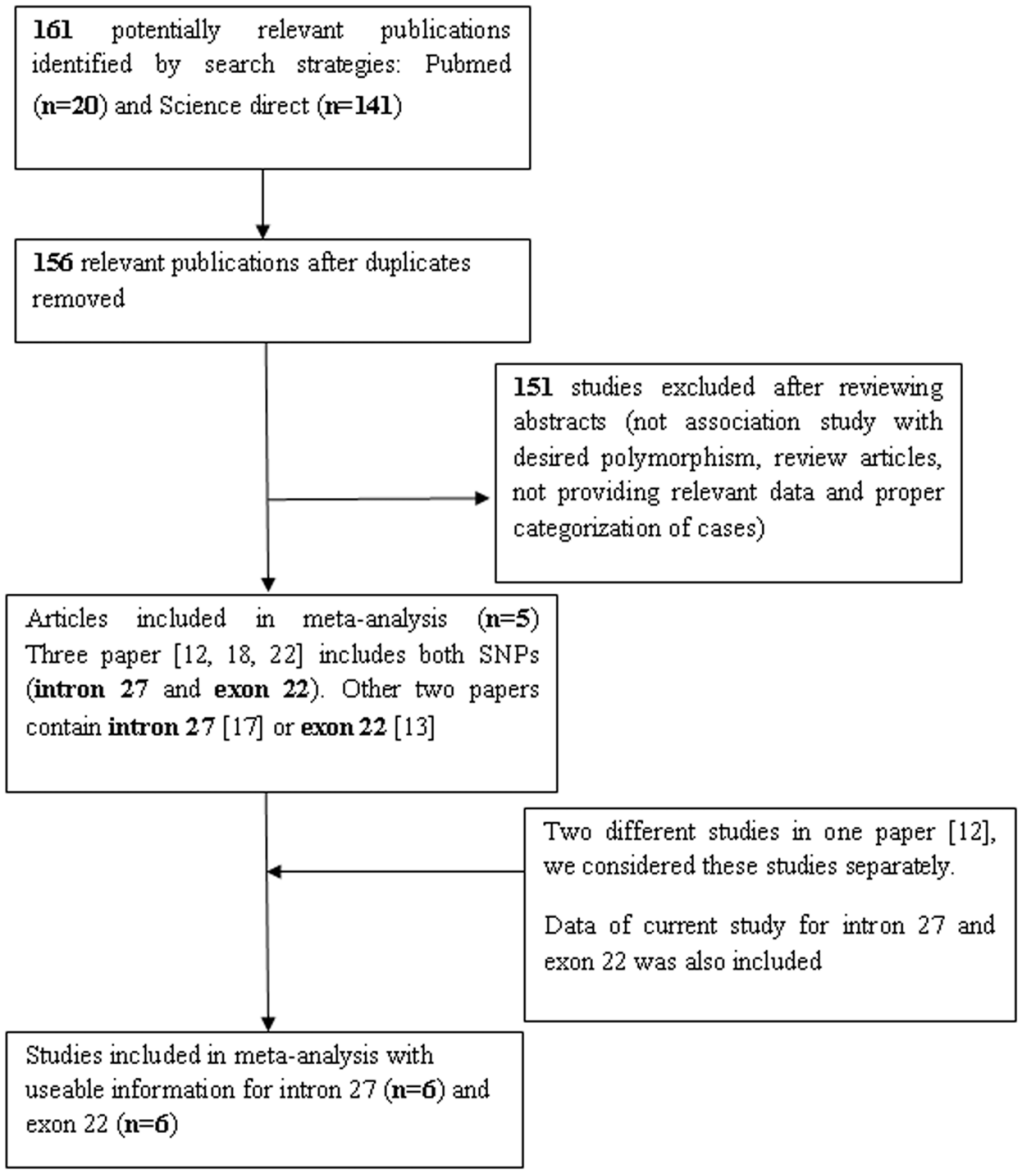
Flow diagramme of identifying potential studies for meta-analysis.

## Results

### Characteristics of Study Participants

A total of 494 *P. falciparum*-infected patients were enrolled in the present study, including 353 severe (SM) and 141 uncomplicated patients (UM). Severe *P. falciparum* malaria patients were further subdivided into cerebral malaria (CM) (n = 97), multi-organ dysfunction (MOD) (n = 129) and non-cerebral severe malaria (NCSM) (n = 127). 100 healthy controls (HC) were included. The difference of mean age between all clinical categories and HC was comparable. Significantly higher levels of haemoglobin were observed in HC than UM and other clinical categories of severe malaria (P<0.0001) (Table 1).

**Table 8:** Statistics to test publication bias and heterogeneity in meta-analysis.

SNP	Comparisons	Egger’s regression analysis			Heterogeneity analysis			Model used for meta-analysis
		Intercept	95% Confidence Interval	P value	Q value	P_heterogeneity_	I^2^ (%)	
exon 22	G vs A	2.39	−3.08 to 7.87	0.29	16.79	0.005	70.23	Random
	GG vs AA	1.81	−2.17 to 5.79	0.27	10.46	0.06	52.23	Random
	AG vs AA	−0.99	−9.79 to 7.79	0.76	33.47	<0.0001	85.06	Random
	GG+AG vs AA	0.56	−3.82 to 4.95	0.73	9.05	0.10	44.76	Fixed
	GG vs AA+AG	1.82	−3.08 to 6.74	0.36	15.00	0.01	66.68	Random
intron 27	T vs A	3.42	−3.87 to 10.00	0.26	34.38	<0.0001	85.45	Random
	TT vs AA	2.85	−3.43 to 9.14	0.27	27.43	<0.0001	81.79	Random
	AT vs AA	0.13	−4.10 to 4.36	0.93	7.98	0.15	37.35	Fixed
	TT+AT vs AA	1.65	−3.47 to 6.78	0.42	16.82	0.005	70.27	Random
	TT vs AT+TT	3.84	−2.02 to 9.91	0.14	26.95	<0.0001	81.45	Random

### CR1 Polymorphisms and ABO Blood Group Distribution in Healthy Controls

To assess the prevalence of CR1 polymorphism (exon 22 and intron 27) in studied population, 100 healthy controls were genotyped by PCR-RFLP and the results are shown in Table-2. For exon 22 polymorphism, frequency of GG genotype was higher (44%) than AG (41%) and AA (15%). Intron 27 heterozygous mutant (AT) was more frequent (51%) in healthy controls than other genotypes: AA (22%) and TT (27%). The distribution of both exon 22 and intron 27 genotypes in healthy controls were in agreement with HWE. Prevalence of ABO blood groups in healthy controls are in accordance to our previous observation [2].

**Figure 2:**
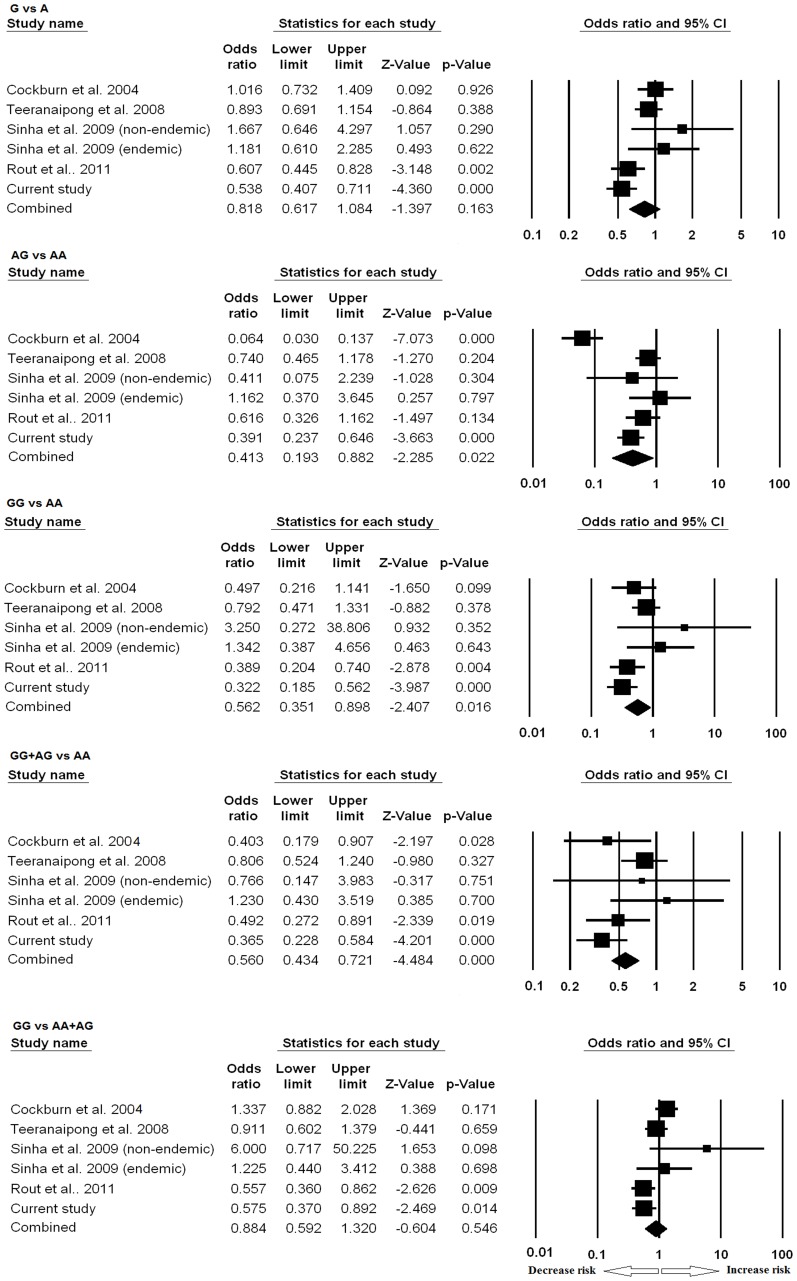
Forest plots of CR1 exon 22 polymorphism in association to severe malaria. Meta-analysis was performed including previous reports and current study by comprehensive meta-analysis software. Random or fixed model of meta-analysis was employed for calculation of the combined effect of all studies. Forest plots evaluating resistance/risk factor of different models to severe malaria are shown.

**Figure 3:**
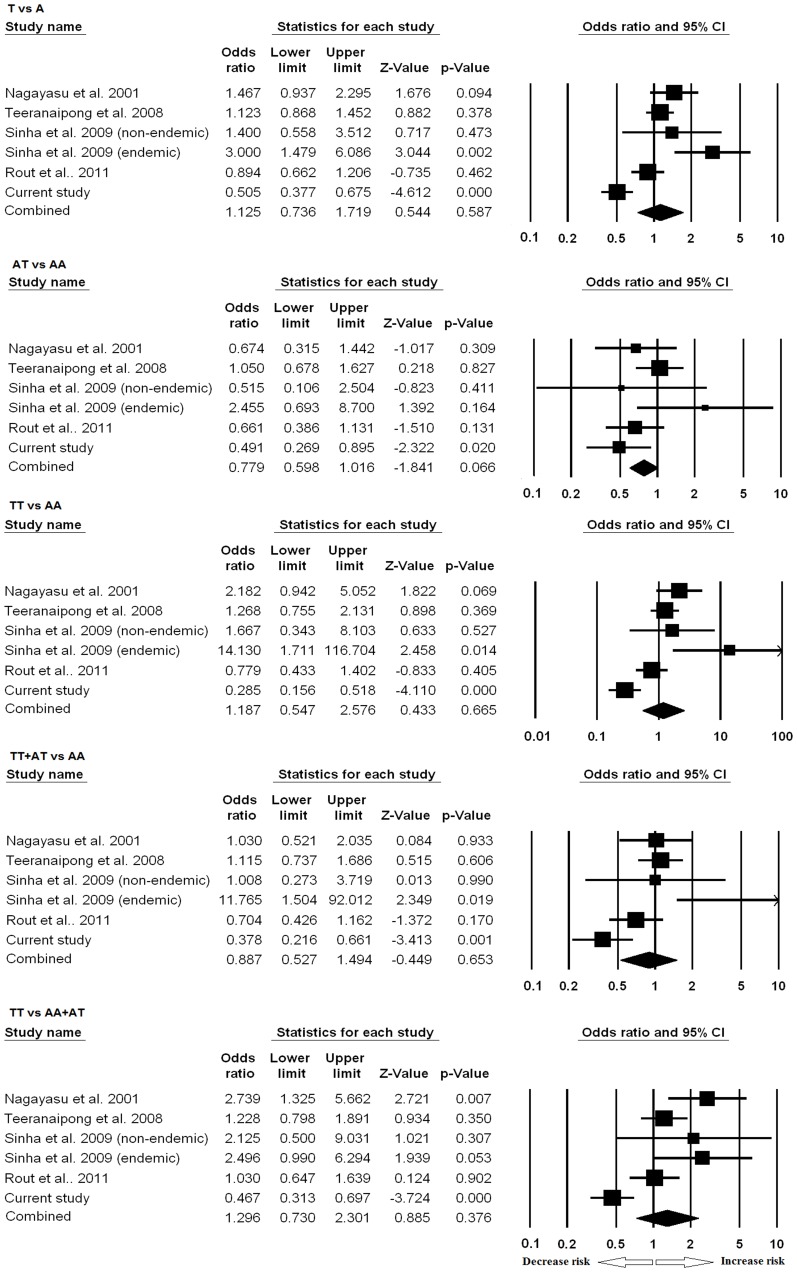
Forest plots of CR1 intron 27 polymorphism in association to severe malaria. Meta-analysis was performed including previous reports and current study by comprehensive meta-analysis software. Random or fixed model of meta-analysis was employed for calculation of the combined effect of all studies. Forest plots evaluating resistance/risk factor of different models to severe malaria are shown.

### Association of CR1 Polymorphisms with Severe Malaria

We used a case control study to examine whether common polymorphism at CR1 gene is associated with severe *P. falciparum* malaria. Two functional CR1 polymorphisms (exon 22 and intron 27) in *P. falciparum* infected subjects were typed and results are shown in Table-2.

For the CR1 exon 22 polymorphism, the frequency of the GG genotype and variant allele G were significantly lower in SM compared to UM (GG: P<0.0001, OR = 0.28; G: P<0.0001, OR = 0.50). The SM patients were further divided into sub-clinical categories and compared to UM. Prevalence of homozygous mutant (GG) and allele G were significantly lower in CM (GG: P<0.0001, OR = 0.21; G: P<0.0001, OR = 0.43) and MOD (GG: P<0.0001, OR = 0.18; G: P<0.0001, OR = 0.40) compared to UM. HC displayed significantly higher frequency of allele ‘G’ and GG genotype than CM and MOD (data not shown). Genotype and allele distribution among NCSM, UM and HC were comparable (Table 2).

For CR1 intron 27 polymorphism, frequency of low CR1 expressing genotype ‘TT’ and allele ‘T’ were significantly higher in UM than SM (TT: P<0.0001, OR = 0.32; T: P<0.0001, OR = 0.53), CM (TT: P<0.0001, OR = 0.18; T: P<0.0001, OR = 0.37) and MOD (P<0.0001, OR = 0.21, 95%CI = 0.30 to 0.60). Furthermore, prevalence of heterozygous (AT) also significantly higher in UM compared to SM (P = 0.0002, OR = 2.55), CM (P<0.0001, OR = 0.25) and MOD (P = 0.0001, OR = 0.32). The genotype and allele distribution of CR1 intron 27 polymorphism were comparable in NCSM, UM and HC (Table 2).

Since both polymorphisms of CR1 were associated with protection against severe malaria, we analysed the distribution of CR1 haplotypes in different clinical categories to study their probable association. The prevalence of haplotype ‘A-A’ was significantly higher in SM (P = 0.0003, OR = 2.35), CM (P = 0.0003, OR = 3.03) and MOD (P = 0.0001, OR = 3.01) compared UM. Distribution of haplotypes in UM and NCSM was comparable (Table 3).

### Association of ABO Blood Group and Severe Malaria

Previously we had demonstrated the association of ABO blood group with severe malaria in Odisha population [2]. In the present report a total of 388 *P. falciparum* infected patients were typed for blood groups comprising of CM (n = 75), MOD (n = 93), NCSM (n = 101) and UM (n = 119). Blood group ‘B’ was significantly higher in SM (P<0.0001, OR = 4.38), CM (P<0.0001, OR = 6.32), MOD (P<0.0001, OR = 4.51) and NCSM (P = 0.0005, OR = 3.31) compared to UM in current study (Table-2) observations corroborating with our previous report [2].

### CR1 Polymorphisms and Prognosis

Since the study revealed a significant association of CR1 polymorphisms (exon 22 and intron 27) with protection against severe malaria, possible role of these variants to the disease outcome was explored. For CR1 exon 22 polymorphism, prevalence of both heterozygous (AG) and homozygous (GG) genotypes were significantly higher in survivors than non-survivors (AG: P = 0.008, OR = 2.63; GG: P = 0.0003, OR = 4.58). For CR1 intron 27 polymorphism, the frequency of AT and TT genotype were also higher in survivors than non-survivors (AT: P = 0.002, OR = 2.92; TT: P = 0.0005, OR = 5.44) suggesting protection against mortality. Interestingly, the mutant alleles of both polymorphisms also showed higher prevalence in survivors (G: P = 0.0001, OR = 2.46; T: P<0.0001, OR = 2.82) (Table-4). We further extended our investigation and analysed distribution of CR1 haplotypes in disease outcome. As shown in Table-5, the frequency of haplotype A-A was significantly higher in patients who died compared to the survivors (P = 0.0007, OR = 0.29). Distributions of other haplotypes were comparable among survivors and non-survivors (Table-5).

We also analysed the prevalence of different blood groups in survivors (Table 4). Although, frequency of blood group ‘B’ was higher in patients who died (57%) compared to those who survived (39%), the difference was statistically insignificant (P = 0.07).

### Association of Combined CR1 Polymorphisms and ABO Blood Group with Severe Malaria

We had earlier demonstrated an independent association of CR1 polymorphisms, haplotypes and ABO blood groups with predisposition to severe *P. falciparum* malaria and prognosis. A combined analysis of these two important factors was done based on the hypothesis that they contribute to rosette formation which has a significant role in the pathogensis of severe malaria.

As shown in Table-6, the prevalence of blood group ‘B’/exon 22(AA)/intron 27(AA) type was higher in SM (P = 0.002, OR = 14.00), CM (P = 0.004, OR = 17.00) and MOD (P = 0.004, OR = 17.00) compared to UM. The distributions of other combined genotypes were comparable among clinical categories.

### Characteristics of Eligible Studies for Meta-analysis

Characteristics of eligible studies are shown in Table-7. There were 161 publications relevant to search key words (Pubmed: 20, Science direct: 141). The selection procedure for appropriate reports is shown in figure 1. A total of 3 studies were examined for association of CR1 polymorphisms (both exon 22 and intron 27) and severe malaria [13], [19], [28]. Other two reports investigated role of intron 27 [18] and exon 22 [14] polymorphism individually. A study by Sinha et al. [13] includes two sets of data for endemic and non-endemic areas and both were included in the meta-analysis. In addition, the data of the present study was also included. Finally, six studies for intron 27, involving 891 severe malaria patients and 772 mild malaria subjects were included. For exon 22, a total of six studies including 1040 severe malaria and 818 mild malaria cases were analysed.

### Publication Bias

Funnel plot and Egger’s test were conducted to assess the publication bias in the reports included for meta-analysis. The shape of funnel plots did not reveal any evidence of asymmetry (Figures not shown). Egger’s test further strengthens statistical evidence of funnel plot symmetry and did not show any evidence of publication bias (Table-8).

### Test of Heterogeneity

Heterogeneity among studies was tested by Q test and I^2^ statistics. Results are shown in Table-8. Heterogeneity was observed in comparison of alleles for both. In addition, genotypes comparison (GG vs AA and AG vs AA) and genetic dominant model for exon 22 polymorphism (GG vs AA+AG) also showed significant variation among studies. Furthermore, dominant (TT vs AT+TT), recessive (TT+AT vs AA) genetic model and genotype (TT vs AA) of intron 27 polymorphism displayed significant variation among studies, thus random effect model was used for calculation of combined OR and 95% CI. Fixed effect model was employed for calculation of pooled OR in recessive genetic model (GG+AG vs AA) for exon 22 and genotype (AT vs AA) comparison for intron 27 polymorphism as they did not show significant heterogeneity.

### Meta-analysis Results

Meta-analysis result of exon 22 polymorphism is shown in Figure 2. Compared to wild-type AA homozygous genotype, the AG heterozygous was significantly associated with protection from SM (P = 0.02, OR = 0.41, 95%CI = 0.19 to 0.88). Similar associations were also observed in dominant genetic model (GG+AG vs AA: P<0.0001, OR = 0.56, 95%CI = 0.43 to 0.72) and homozygous genotype comparison (GG vs AA: P = 0.01, OR = 0.56, 95%CI = 0.35 to 0.89). The allele contrast (G vs A) and recessive genetic model (GG vs AA+AG) did not show any association.

Meta-analysis of CR1 intron 27 polymorphism is shown in Figure 3. None of the models was found to be significantly different among SM and UM.

## Discussion

In this case-control study we investigated the role of common CR1 variants and blood groups in *P. falciparum* malaria in an endemic region of Odisha, India. Our data suggest that mutants of CR1 polymorphims (exon 22 and intron 27) and blood group ‘O’ protect *P. falciparum* infected subjects from severe disease and mortality. Furthermore, a meta-analysis revealed a significant association of exon 22 polymorphism with severe malaria in most endemic regions of the world.

Malaria has a strong selective pressure on human genome. In endemic areas, it positively affects blood cell polymorphisms which protects against *P. falciparum* infection [38]. In accordance with our earlier report [2], the blood group ‘O’ was more prevalent in studied population, which is protected. This is the first study that clearly shows an association of CR1 variants with protection from malarial death. This finding probably explains and is in accordance with higher prevalence of CR1 intron 27 minor allele (T) in various malaria endemic areas like Thailand [18] and India [13], [19]. In malaria non endemic area, there is a lower frequency of allele ‘T’ [14], [39]. The distribution of other CR1 variant (exon 22) also showed similar pattern: higher prevalence of minor allele (G) in endemic population Papua New Guinea [14], Thailand [18], India [13], [19] and low prevalence in non endemic areas [13], [14]. Furthermore, in the current study minor allele frequency of CR1 intron 27 and exon 22 were 52.5% and 64.5% respectively in healthy controls. Collectively, these observations strongly indicate a positive selection of low CR1 expressing allele in malaria endemic areas. Association of common functional polymorphisms at CR1 genes with severe malaria have been studied in various populations [13], [14], [18], [19]. Conclusions from previous reports on the role of intron 27 and exon 22 polymorphisms in severe malaria risk/resistance remain conflicting. These inconsistencies could be attributed to small sample size, non categorization of severe malaria into sub categories and areas of different endemicity In the current study, we enrolled a large number of patients with defined clinical categorisation from an endemic area and studied the association of CR1 polymorphisms with *P. falciparum* infection. Data of the present study showed significant association of CR1 (intron 27 and exon 22) mutants with protection against CM and MOD. In an earlier report association of CR1 variants with CM but not with MOD had been demonstrated [19]. This disagreement could be attributed to small number of MOD cases assessed in their series.

CR1 intron 27 and exon 22 variants are associated with severe *P. falciparum* malarin in different endemic geographical regions [13], [14], [18], [19]. Both intron 27 and exon 22 polymorphisms correlate with CR1 expression on RBCs [13], [19]. Mechanism by which intron 27 may affect CR1 expression is unknown. The other SNP lies in short consensus repeats-19 of CR1 molecule which could affect the structure and stability of CR1. The results of the present study also highlight the effect of combined CR1 variants and blood groups with regard to severe disease. Distribution of ‘B’ blood group and wild type of CR1 polymorphism (intron 27: AA/exon 22: AA) were significantly associated with severe malaria (OR = 14). Patients with combined genotypes ‘B’/AA/AA had 17 fold higher chances of developing CM or MOD. In the studied population we had demonstrated the association of blood group ‘B’ with severe malaria [2]. Patients with B/AA/AA genotype perhaps have synergistic effect with regard to rosetting.

Meta-analysis is a powerful tool which combines finding of independent similar studies and derive a definitive conclusion [40]. Several studies have investigated the association of common functional variants of CR1 with severe malaria. Current meta-analysis revealed a significant association of CR1 (exon 22) variant with protection from severe disease. On the contrary, the meta-analysis failed to show an association between intron 27 polymorphism with severe malaria. These findings indicate inter-study variations which could be multi-factorial. Malaria endemicity affects the erythrocyte CR1 expressions and its function. In non-endemic population the concept of CR1 variants and disease susceptibility does not appear to hold true [13]. The mechanism related to polymorphisms at CR1 gene and disease severity remains ill-defined although increased rosetting is believed to be a factor. There is also a discordance between the quantitative expression of CR1 and its functionality [10]. Despite these limitations in understanding of cause effect relationship, CR1 polymorphisms and ABO blood group system appear to affect the outcome of *P.falciparum* infection in malaria endemic areas.

## Supporting Information

Supplement S1 **PRISMA 2009 checklist.**
(DOC)
